# Supplementary material for: Chromothripsis during telomere crisis is independent of NHEJ, and consistent with a replicative origin
Source: Genome Res. 2019 May;29(5):737–49. doi: 10.1101/gr.240705.118 (PMC6499312; doi:10.1101/gr.240705.118)
Supplement: Supplemental Material [file supp_gr.240705.118_Supplemental_file_1.zip › contigs/annotated_contigs/DB93/contig.2.DB93_length_366_mean_cov_6.89890710383.docx]

**DB93_length_366_mean_cov_6.89890710383**

```
TACCTTTAATATATTTTCAGTTTTAAGATTGGCTGGCCAGGGGCGGTGCCTCATGCCTGTAATCCCAGCACTTTGGGAGGCCGAGGCAG
 >chr3:47779358-47779486 - E=1e-56 p=0e+00
GGCGGATCACGAGGTCAGGAGATCGAGACCATCCTGGCT|GATAGCT|CTGATCAGGCTGGACGCGGTGGCTCACACCTGTAATCCCAG
                                               >chr3:47760901-47761132 + E=1e-127
CACTTTGAGAAGCCAAGGTGGGTGGATCACCTGAGGTCAGGAGTGCAAGACCAGCCTGACCAGCATGGTGAAACCCCGTCTCTACTAAA

AATACAAAAATTAGCCAGGCATGGTTGGGGGGCATCTGTAATCCCAGCTACTAGGAAGGATGGAGGCAGGAGAATCACTTGAACCCAGG

AGGTGGAGGTTG
```
